# A Type 2 Diabetes Prevention Website for African Americans, Caucasians, and Mexican Americans: Formative Evaluation

**DOI:** 10.2196/resprot.2573

**Published:** 2013-07-11

**Authors:** Belinda Reininger, Laurel Person Mecca, Kendra M Stine, Kevan Schultz, Luke Ling, David Halpern

**Affiliations:** ^1^Associate ProfessorHealth Promotion and Behavioral SciencesUniversity of Texas School of Public Health, Brownsville Regional CampusBrownsville, TXUnited States; ^2^Assistant Director & Senior Research Specialist Qualitative Data Analysis ProgramUniversity Center for Social and Urban ResearchUniversity of PittsburghPittsburgh, PAUnited States; ^3^Senior Research Assistant, Non-ClinicalHealth Promotion and Behavioral SciencesUniversity of Texas School of Public Health, Brownsville Regional CampusBrownsville, TXUnited States; ^4^Research Specialist, Qualitative Data Analysis ProgramUniversity Center for Social and Urban ResearchUniversity of PittsburghPittsburgh, PAUnited States; ^5^Web Technical SpecialistCancer Center Information Services DivisionUniversity of Pittsburgh Medical CenterPittsburgh, PAUnited States

**Keywords:** diabetes, Internet, Mexican-Americans, African Americans, socioeconomic status, dietary intake, physical activity, health literacy, website

## Abstract

**Background:**

The majority of Americans now access the Internet, thereby expanding prospects for Web-based health-related education and intervention. However, there remains a digital divide among those with lower income and education, and among Spanish-speaking populations in the United States. Additional concerns are the low eHealth literacy rate among these populations and their interest in Internet-delivered interventions with these components. Given these factors, combined with the prevalence of type 2 diabetes among low socioeconomic status and Spanish-speaking Americans, strides need to be taken to reach these populations with online tools for diabetes prevention and management that are at once accessible and efficacious.

**Objective:**

Using a formative evaluation of an eHealth diabetes prevention and control website, we tested the extent to which African Americans, Caucasians, and Mexican Americans at risk for type 2 diabetes gained knowledge and intended to modify their dietary intake and physical activity subsequent to viewing the website. We also examined their general Internet use patterns related to type 2 diabetes.

**Methods:**

A mixed methods approach was undertaken. The diabetes prevention and control website provided educational and behavioral change information in English and Spanish. For this study, eligible participants (1) completed a prequantitative survey, (2) interacted with the website, (3) completed a qualitative interview, and (4) completed a postquantitative survey.

**Results:**

After finding a significant differences in posttest diabetes knowledge scores (*P*<.001), a regression analysis controlling for pretest score, health literacy, ethnicity, Transtheoretical Model Stage for exercise and fruit and vegetable consumption, and Internet literacy was conducted. Internet literacy score (*P*=.04) and fruit and vegetable consumption stage (*P*<.001) were significantly associated with posttest scores indicating that those in precontemplation stage and with low Internet literacy scores were less likely to show improved diabetes knowledge scores. We found significant difference in posttest intention to eat a healthy diet each day in the next 2 months after controlling for pretest score, health literacy, ethnicity, Transtheoretical Model Stage for fruit and vegetable consumption and Internet literacy. Those in the Action stage of the Transtheoretical model for exercise were significantly less likely (*P*=.023) to improve the posttest score for intention to eat a healthy diet compared to those in the Preparation stage for exercise. We also found that health information is sought commonly across ethnic groups, but that diabetes-related information is less commonly sought even among those at risk. Other specific ethnic usage patterns were identified in the qualitative data including content sought on Web searches and technology used to access the Internet.

**Conclusions:**

This study provides in-depth qualitative insight into the seeking, access, and use of Web-based health information across three ethnic groups in two languages. Additionally, it provides evidence from pre-post measures of exposure to Web-based health content and related changes in diabetes knowledge and intention to eat a healthy diet.

## Introduction

The ubiquity of the Internet presents opportunities to equalize access to and usage of diabetes prevention and control information, particularly for health disparate populations including African American and Mexican American populations whose incidence rates of diabetes exceed Caucasians [1]. Obesity resulting from excess calorie intake and physical inactivity over time is associated with diabetes [2]. In the United States, 44.1% of African Americans, 32.4% of Caucasians, and 40.4% of Mexican Americans are classified as obese [3,4]. None of these populations meets federal nutrition [5-7] or activity guidelines consistently. For example, only 11% of the US population met fruit and vegetable consumption guidelines [5] and fewer than 5% met physical activity recommendations [8]. Addressing these behaviors through the Internet to increase healthful nutrition and energy expenditure may contribute to diabetes prevention and control strategies.

Vigilance on issues of unequal access to eHealth [9,10] has now shown that the majority of Americans access the Internet [11]. While there has been and still is a digital divide among those with lower income and education and Spanish-speaking populations in the United States, even these populations are reporting more access to the Internet because of expanded technologies, particularly mobile devices [11].

Today, an additional concern is low eHealth literacy [12,13], which means an individual may have difficulty not only seeking, finding, appraising, and understanding electronic health information, they may also not have the knowledge to apply the information to solve the health problem [13]. The design of diabetes prevention and control websites to accommodate low eHealth literacy abilities with particular attention on learning and usability issues [14] across ethnic populations is an area of growing importance.

There is mounting interest in eHealth/Internet-delivered interventions [15-17], especially studies testing the efficacy of websites singularly or in combination with other intervention components. We conducted a formative evaluation of an eHealth diabetes prevention and control website, including content related to nutrition and physical activity, available in Spanish and English with African Americans, Caucasians, and Mexican Americans at risk for diabetes. A mixed methods approach was taken to examine learning and usability features of the website. We examined the participants’ general Internet use patterns related to type 2 diabetes. We hypothesized that our participants would gain knowledge and intend to modify their dietary intake and physical activity subsequent to viewing the website. Here, we report both results describing the Internet-based, health information seeking, access, and use by ethnic group, and pre-post measures of exposure to health content on a website, as well as related changes in knowledge and intention to engage in preventive behaviors.

## Methods

### Website Development

The Pittsburgh Regional Initiative for Diabetes Education (PRIDE) initially created a website with content composed by experts in the fields of diabetes education and prevention at the University of Pittsburgh Diabetes Institute (PrideofPA website) [18]. The website provided educational and behavioral change information for those at risk for or diagnosed with type 2 diabetes. Features of the site include videos on topics such as physical activity and nutrition, medications, social support, and a local map pinpointing nearby Diabetes Self-Management Education locations. In addition, this site has a diabetes risk calculator to help people determine their potential risk for diabetes. The risk calculator is based on risk factors identified by the American Diabetes Association (ADA) and allows individuals to answer a few simple questions to determine their risk for type 2 diabetes. The website directs people to be screened and talk with a medical provider. See Multimedia Appendices 1-3 for examples of website content.

The website content and risk calculator were also translated into Spanish by professional translators specializing in Mexican-origin Spanish and culturally relevant examples were added to content and videos. Feedback from Mexican American end users was obtained through cognitive interviews (TSSC website) [19]. The resulting website available in English or Spanish was then the focus of a formative evaluation process whereby eligible participants (1) completed a prequantitative survey, (2) interacted with the website, (3) completed a qualitative interview, and (4) completed a postquantitative survey.

### Participants

A total of 71 adults at risk for diabetes because of overweight or obesity status and/or family history of diabetes were recruited across African American, Caucasian, and Mexican American populations to participate in one-on-one interviews, in order to assess whether and in what ways the website and diabetes risk calculator were appropriate for moderate- and high-risk populations. Participants in southwestern Pennsylvania (PA) were asked to review an English language version of the website and risk calculator and were either African American (n=19) or Caucasian (n=27), while the Mexican American participants (n=25) were from Brownsville, Texas (TX), and were asked to review a Spanish-language version of the website.

### Recruitment

Recruitment and data collection were carried out between summer 2011 and spring 2012. In either study site (PA or TX), participants were recruited via local universities, churches, physician offices, school organizations, Internet sites, and community organizations/events. The recruitment process was the same across the three populations. Caucasian, African American, or Mexican American adults aged between 18-54 years, who read and speak English or Spanish, with a body mass index (BMI) greater than 24 and/or who were at risk for diabetes by family history, and had an email address, were considered eligible for participation. Individuals ineligible for the study included women who were pregnant, those currently diagnosed with diabetes, those unwilling to access the Internet for health information, and those who were in the Maintenance stage for fruit and vegetable consumption or exercise according to the Transtheoretical Model [20]. A staff member or project volunteer provided an overview of the project, explained inclusion/exclusion criteria, and concluded with a request for volunteers to participate in the study, including the first step of completing the eligibility screening. Separately, fliers were distributed that summarized key points of the project and included a phone number to reach a study contact.

### Screening Process

Recruited participants were screened for eligibility via an online battery of tools hosted by SurveyMonkey. All participants who completed the eligibility screening tools received a US$10 incentive regardless of their study eligibility.

Participants were asked to answer the following:

1. Would you ever consider using the Internet to search for health information? Participants who answered “no” to this question were disqualified from participation in the study.
2. Do you have a family member who has diabetes (mother, father, or sibling)? Participants who answered “yes” to this question qualified as at risk for diabetes for the purposes of this study.
3. Self-reported height and weight. These data were used to calculate BMI. Participants categorized as overweight or obese and at risk for diabetes were deemed eligible for the study.
4. Internet Literacy Assessment. A literacy tool used to characterize participants as low, moderate, or high Internet users [21] but was not used for eligibility criteria.
5. Transtheoretical Model Staging Algorithm Pretest. This assessed participants’ readiness to meet guidelines for physical activity and fruit/vegetable consumption [20] with those in maintenance being excluded from the study.

Participants who qualified as eligible and were interested in enrolling in the study went on to complete an additional two questionnaires: (1) Diabetes Knowledge Questionnaire Pretest, which was a 25-question, self-administered questionnaire that assessed the participant’s diabetes knowledge [22,23], and (2) Theory of Planned Behavior Questionnaire Pretest, which was a 56-question, self-administered questionnaire designed to assess the participant’s intentions to change or modify their behavior [24].

### Interviews

Participants were contacted via phone by a study representative to schedule an in-person study session, to be conducted at the University of Pittsburgh Medical Center or the University of Texas School of Public Health Brownsville Regional Campus. The sessions consistently lasted approximately 90 minutes and were conducted in the participant’s native language (English or Spanish). First, informed consent was obtained from participants. Second, participants completed the Short Test of Functional Health Literacy in Adults [25].

Next, participants were introduced to the website and instructed that they had 20 minutes alone to explore it. At the conclusion of the 20 minutes, the study representative returned to the room and asked participants to complete three guided tasks on the site about diabetes: (1) to identify two things they learned about being at risk for type 2 diabetes, and (2) to assess in a real-life scenario how they would apply the information. Participants were given 15 minutes to complete these tasks.

Upon completion, trained study staff performed a semistructured interview to assess the participant’s overall thoughts about the website. The audio-recorded interviews ranged from 10-60 minutes in duration, with a mean of 35 minutes. Interview questions inquired about the participant’s diabetes risk test results, past experiences searching for health information online, perceptions of the website, and motivations and barriers to exercising and making healthy food choices.

To conclude the session, participants were asked to complete posttest assessments on the following three surveys: Diabetes Knowledge Questionnaire, Transtheoretical Model Staging Algorithm, and Theory of Planned Behavior Questionnaire.

### Qualitative Analysis

Audio recordings of the semistructured interviews were transcribed verbatim. Qualitative analysis of the transcripts commenced with the full research team identifying an initial list of codes based on the questions included in the interview guide. This list of codes was then expanded upon and refined through four rounds of test coding (13/71 or 18.3% of the transcripts were double coded) using ATLAS.ti 6.0, a computer-assisted qualitative data analysis software program. In the first round of test coding, 2 coders (one from each study site) each analyzed selected participant transcripts. Using EUSEBIUS, a software package developed in-house at the Qualitative Data Analysis Program, this first round of coding agreement was adjudicated by the full research team and a Fleiss’ kappa was calculated to measure intercoder agreement. The kappa statistics served to guide codebook modifications. The codebook was finalized once it was deemed to effectively capture the key themes emerging from the interviews with relevance to the project’s research questions. The 2 initial coders from each site also served as adjudicators and coaches for the 6 other coders to ensure consistency within and across the sites.

Once the coding was completed, members of the research team reviewed the coded data to identify the dominant themes. Further, distinctions were made concerning viewpoints shared by participants from all three ethnic groups and those that differed according to ethnicity. Although the quotes for the Mexican American participants in this study are presented in English, the interviews were conducted in Spanish.

### Quantitative Analysis

A comprehensive dataset was established for each participant including their SurveyMonkey data and the paper-pencil data from posttest. Double data entry was conducted on any paper-pencil collected data to ensure accuracy. For univariate analysis, a *t* test or its nonparametric counterpart, a Wilcoxon rank-sum test, was used. For multivariate analysis, a linear regression was conducted. A significance level of 0.05 was used in the analyses to determine significance.

## Results

### Descriptive and Qualitative Results

The sample for this study included African American, Caucasian, and Mexican American adults, primarily females, with some college education, and making under US$40,000/year (Table 1).

We report on Internet usage for health seeking by ethnic group, related generally to health and specifically to diabetes, and on whether content on a website related to diabetes increased knowledge about and intention to engage in preventive behaviors. We have summarized qualitative and quantitative findings related to these topics.

**Table 1:** Demographics of sample (N=71).

	Gender		Education level		Household income	
Ethnic group	Male % (n)	Female % (n)	<College % (n)	Some college+ % (n)	<$40,000 % (n)	>$40,000 % (n)
African American (n=19)	15.8 (3)	84.2 (16)	10.5 (2)	89.5 (17)	78.9 (15)	21.1 (4)
Caucasian (n=27)	14.8 (4)	85.2 (23)	0.0 (0)	100.0 (27)	13.24 (9)	25.00^a^ (17)
Mexican American (n=25)	16.0 (4)	84.0 (21)	24.0 (6)	76.0 (19)	87.0 (20)	13.0 ^a^ (3)
Total (71)	15.5 (11)	84.5 (60)	11.27 (8)	88.73 (63)	64.7 (44)	35.3 ^a^ (24)

^a^Participants refused to answer.

#### Internet Searching Theme: Seeking Internet-Based Health Information Is Normative

Participants described regular interactions with Web-based health information. Searches for online health information occurred either as a routine part of their life, or as an intermittent targeted search activity, or a combination of both (Table 2):

1. *Habitual Searching*: Participants described regularly seeking online health information as part of a daily or regular pattern. They also indicated that their searches covered a wide variety of health topics. Participants frequently used the term “To Google it” when asked about online searching methods, which reinforces not only their familiarity with searching for information online but the extent to which Google’s search engine has permeated our culture.
2. *Intermittent Health Information Searching:* Participants also discussed searching for online health information when particular questions arose or circumstances dictated the need for this information.
3. *Habitual and Intermittent Health Information Searching:* Some participants discussed a combination of patterns for searching for health information. They may regularly be online learning about health topics (eg, a chronic health condition they have), but then intermittently “look into” certain topics.

Studies focused on the digital divide specific to online health-related information seeking indicate that the propensity to search online for health information is linked most strongly with education level and Internet access. That is to say that the higher the education level and the greater the Internet access, the more likely one is to search online for health information. Differences in the rate of electronic health information seeking between ethnic groups do exist but to a lesser extent [26,27]. In our sample, there were no marked differences by ethnic group in the description of the frequency of searches. However, while all participants had indicated a willingness to search for health information as criteria for inclusion in the study, 2 Mexican American participants interviewed commented that they had never searched for health information online, while all African American and Caucasian participants indicated some past history with searching online.

**Table 2:** Quotes from frequency theme: searching for health information is commonplace.

Participant ethnicity	Habitual searching	Intermittent searching
African American	A lot, couple times a week. Not necessarily diabetes, but always about health. Always about eating and exercising, always.	Mmm…couple times a year, just depending on what’s going on in the family.
Caucasian	Weekly. Yes. Pretty much general definition, you know then that kind of segues into symptoms. You know, not drilling down into management, but, risk factors, symptoms, starting there.	My daughter has a lot of medical problems, so I do use the Internet a lot. Basically I would just go into Google or Yahoo, type in the basic thing that I was looking for. Sometimes it would be a little overwhelming because you have all these links and you don’t know exactly where to go...For example, she has asthma, so I would type in things, like…ways to make it more tolerable, or ways to treat, alternative treatment methods, things like that. Just like a general term. On average probably two to four times a month maybe, depending on what I’m going through at the time. If I’m sick or she’s sick.
Caucasian	Quite a bit, especially lately. You know, a couple times a week at least, you know, at various times through the day, and so it’s been frequent lately.	
Mexican American	There are times that people tell me “hey, there is a website.” Or some time ago, people told me that there was a talk about stem cells, that they may help diabetes. That is when I go in and look…I am not on the computer all day because I am a stay home mom. Whenever I have a chance I sit down and read. Generally it is once a week…and I also get my daughter involved.	I would say like every 2 weeks, when I get a chance I go and…search this and that.
Mexican American	Two or three days of the week or more when I feel like working at the computer.	

#### Content Theme: Searching for a Broad Spectrum of Health Content

Participants described searching for health information either to increase their general knowledge on a condition or health topic, or in a more targeted manner to understand ailments and symptoms (Table 3). The reasons for these searches were often imbedded in personal, friend, or family need for information.

##### Searches for Health Conditions or General Health Topics

The spectrum of health content searched for by participants ranged widely. Topics such as men’s health, cancer, mental health, various chronic conditions (multiple sclerosis, polycystic ovary syndrome, autoimmune conditions), flu symptoms, sexually transmitted diseases, concussions, food allergies, caloric intake, hypoglycemia/hyperglycemia, thyroid disorders, health care provider information, and side effects of medications were examples of health information that had been previously sought. Participants were interested in learning more about health issues they or family/ friends were experiencing or health issues they did not understand.

Participants across all three ethnic groups described searching for online health information for themselves; however, it was less common for African American participants to describe searching for health information for family and friends as compared to Caucasian and Mexican American participants.

##### Searches to Understand Symptoms

Participants searched for health information to understand, diagnose, or treat symptoms either they or someone they cared for was experiencing. Symptoms such as cough, aches/pains, asthma, constipation, and H1N1 symptoms were specifically mentioned as reasons to search.

Also, participants from all three ethnic groups commonly mentioned searching for symptoms, with African Americans discussing this search approach slightly less often than Caucasians and Mexican Americans. However, there were no marked differences by ethnic group in how the searches were conducted or approached.

African American participants discussed looking for health information regarding weight status, desire to lose weight, and finding healthier food choices (Table 4), whereas other ethnic groups did not discuss this content.

### Searching for Diabetes-Related Information Specifically

Participants were asked about how they interacted in the past with health information websites of their own choosing specifically on the topic of diabetes.

#### Diabetes Information Theme: Searching for Diabetes Information Online Is Not Universal

We found that of the 71 interviews conducted, 47 (66%) of participants indicated they had searched for diabetes information though there was a range of topics discussed and a difference in depth of search (Table 5). Those participants who had family or friends with diabetes discussed more in depth searches. There were no ethnic differences in the discussion of searching for diabetes information online.

Searching the Internet for any type of health information is commonplace across the ethnic groups and for many of our participants is a regular part of their daily and weekly routines. Diabetes-related online information had previously been sought by 47 of 71 participants but all were deemed at risk for diabetes based on study screening criteria. The participants indicated that they searched for information for themselves regarding symptoms of diabetes and for loved ones with diabetes regarding their dietary intake and medications.

**Table 3:** Content theme: Searching for a broad spectrum of health content.

Participant Ethnicity	Searching for health conditions or general health topics	Searching as a reaction to symptoms
African American	Everything. Literally. But strangely, I didn’t do diabetes. Cancer…Everyone—it seems like everyone’s dying from cancer, heart disease. I mean, it’s kind of like the same thing, and I always thought, food was our trigger, like one of our main triggers, and how we’re eating.	I usually will go on Google, and whatever I think the ailment would be, I’ll just type that in and see what comes up. I’ll go to various websites, but it’s hard when you’re looking up information online ’cause not a lot of the sites are trustworthy. You have to kind of find a way to filter out the good sites from the bad ones. Find out what information is actually useful and what could be discarded…Even if I’m sniffling and sneezing, I’m like, “Ugh, this might be a cold.” I’ll type in “common cold” or “swine flu.”
Caucasian	I would say maybe, maybe once a month. I mean it possibly could be more. Like if I‘m on, like MSN website, MSN.com, and you know they’re advertising something that has to do with health, I might click on it. Or, if I want to look into something…then it might be more.	Anytime I think I’m sick, since I don’t have health insurance, I try to find a website. It will tell me “Well, if you have this and this, it might be this.” I can kind of get a feel for if it’s the flu or something like that…Recently, my girlfriend has some mental health issues, so I’ve been looking up those kind of things on WebMD or different websites like that. That’s the only one I can think of off-hand now. They’ve just been really unhelpful.
Mexican American	Yes, I have searched for cholesterol because that is what always worries me a little bit more, right? For the same reason, because of family issues. I have a grandfather who had a heart attack, and an aunt who had a stroke. So then you focus on cholesterol.	So, there specifically is what most interests me. Since these two had breast cancer, that’s where I focus more to see what risks I have.

**Table 4:** Content theme: Additional health information sought by African American.

Participant ethnicity	Searching for health information about weight loss, dietary intake
African American	Well, weight, you know. What causes the weight, calories intake. I’m still trying to learn about that, what the calories are, and what would be right for me to have in a day. They say women should have no more than 2000 calories—I think it’s 2000, or 1500 to 2000 calories—but what would that consist of, you know? Like what would I have in the morning? Would I have 300 or 400 calories in the morning, and maybe 200 calories for that type of calorie? I’m kind of confused when it comes to how much I should be eating.
African American	It’s always been easy for me to find information. I’m good with computers and it’s just easy. All you have to do is just put in a name and you can just find whatever you want to find…I always look at things about weight. And about food. I always look for weight, food, and exercise. Even in stores I always read labels.

**Table 5:** Searching for diabetes information online is not universal.

Participant ethnicity	Previously searched for diabetes-related information	Has never searched
African American	More so…yeah, my doctor had mentioned something about a drug that she could possibly give me if my blood sugar didn’t improve, so I looked that drug up.	No. I never looked.
Caucasian	Yes. I actually did look for it a couple other times because I was thinking that I was. I just actually remember this because my friend said that she felt that she was experiencing symptoms of diabetes…being thirsty often. And so I remember I Googled it, I was like, “oh, I’m thirsty a lot, too,” and, I was like “I do eat a lot of sugar, so, and it’s in my family.” I went and just looked up about diabetes. Yeah, I was looking for the symptoms, because I think to be diagnosed you have to go to your doctor and have blood work done.	No. For some reason diabetes is [chuckles] not really one of those. I know of some people with diabetes, and it’s not like as serious...like they’re living with it, so.
Mexican American	It is important that she [daughter] sees how the whole system works and how diabetes affects her as well. Very important for her to know. And I also investigate a lot about carbohydrates, because our doctor talks a lot about the importance of the carbohydrates diabetics need to take. Then there are times when we do not know the portions, the foods, the carbohydrates.	No, to tell you the truth because I was never at risk. I really didn’t worry a lot for that. I worried more about triglycerides or something that I’m at risk for.

### Environment for Accessing Online Health Information

#### Access Theme: Utilizing Various Locations and Technologies to Access Online Health Information

Participants also reported conducting online searches in various locations such as their home, place of work, and the library. The most popular location for searching for online health information by all ethnic groups was the home. A range of technology was used including desktops, laptops, tablets, and phones (Table 6).

Ethnic groups differed by location and type of technology used. More Mexican Americans participants accessed Web-based health information on desktop computers, whereas Caucasian participants tended to access Web-based health information more often on phones, laptop computers, or other mobile devices. African American participants also indicated using public facilities such as libraries for Internet searches more often than the other two ethnic groups.

#### Reacting to Risk Score Theme: Features of Website Can Elicit Emotional Reaction

Participants were asked for their opinions on a diabetes risk calculator that was part of the website. The calculator assessed participants’ risk through a series of questions based on the ADA risk factors regarding genetic predisposition to diabetes, levels of physical activity, age, and BMI. Based on their responses, participants received a low, moderate, or high risk score for diabetes.

All but 5 participants received a risk score of either high or moderate. More participants reacted with surprise about a high or moderate score than did those who did not find these scores surprising. Participants who received a low risk score did not express surprise but rather emotions such as relief and happiness. Some of these low-risk participants had made behavioral changes to decrease their risk of diabetes and those behavioral changes were reinforced by the low-risk categorization. Participants reacted differently to the moderate or high risk scores, where some said they would change their behaviors while others did not indicate such changes would be occurring. Ethnic differences among these reactions or indications of intention to change behavior were not found. Quotations representing these themes are presented in Multimedia Appendix 4.

The designers of the website included a risk calculator to provide information about individual risk for diabetes in a manner that would be educational and could create an emotional reaction as a catalyst for actions including being tested, more in-depth information searching, and preventive behavior change. Based on the participants’ reactions of surprise or relief, it appears that the risk score calculator does elicit an emotional reaction. However, only some participants expressed motivation to make changes in their behavior, including seeking support from physicians and recommitting to exercise routines. Other participants reacted to the risk score without statements indicating commitment to change behaviors. For many, but not all, the risk score calculator appears to be an appropriate, thought-provoking, and emotion-provoking element on the website. Furthermore, the themes associated with the risk score calculator do not show patterns by ethnic group.

#### Returning to Website Theme: Indication of Return to Website Likely

In the present study, return use of the website was not tracked over time, but unlike similar studies that only focused on assessing the feasibility/desirability of the website without going forward with full-scale use of the website [28,29], interviewers did inquire about the likelihood of the participants to return to use the website in the future. The overall expected return rate was 81%. We examined the results by ethnic group and found that the predicted return rates for Caucasians were somewhat lower than for other participants: African American 82% (16/19), Caucasian 70% (19/27), and Mexican American 91% (23/25).

### Quantitative Results

Quantitative surveys were administered to participants in this study as a pretest (as part of the enrollment procedure) and as a posttest (after exposure to the website and qualitative interview). We examined whether improvement occurred in participants’ diabetes knowledge after using the website. After bivariate analysis indicated significant mean score differences in diabetes knowledge from pre- to posttest (*P*≤.001), a regression analysis was conducted with diabetes knowledge post score as the dependent variable, controlling for several independent variables (Table 7).

We found significant differences in posttest diabetes knowledge scores after controlling for pre-test score, health literacy, ethnicity, Transtheoretical Model Stage for exercise and fruit and vegetable consumption, and Internet literacy. Internet literacy score, and fruit and vegetable consumption stage were significantly associated with posttest scores indicating that those in pre-contemplation stage and with low Internet literacy scores were less likely to show improved diabetes knowledge scores.

We also examined participants’ intention to be physically active or to eat healthy after viewing the website. We examined posttest intention scores after controlling for pretest intention, health literacy, ethnicity, Transtheoretical Model Stage for exercise and fruit and vegetable consumption, and Internet literacy. We found no significant differences in intention to be physically active. We did, however, find in the bivariate analysis a significant difference in intention to eat a healthy diet each day in the next 2 months from pre- to posttest (*P=*.002).

Based on these findings, we conducted a regression model examining the intention to eat a healthy diet as the dependent variable and pretest intention score, health literacy, ethnicity, Transtheoretical Model Stage for exercise and fruit and vegetable consumption, and Internet literacy as independent variables (Table 8).

All participants were exposed to the website between pre- and posttest administration. We found significant difference in posttest intention to eat a healthy diet each day in the next 2 months after controlling for pretest score, health literacy, ethnicity, Transtheoretical Model Stage for fruit and vegetable consumption, and Internet literacy. Those in the Action stage of the Transtheoretical model for exercise were significantly less likely to improve the posttest score for intention to eat healthy diet compared to those in the Preparation stage for exercise.

**Table 6:** Accessing online health information.

Participant ethnicity	Utilizing various locations and technologies to access online health information
African American	It could be on a laptop at home, my netbook at home, the desktops in the library, and I’ve even done searching on my smartphone.
Caucasian	Yeah, I don’t do phone. I don’t even know what they’re called anymore. [laughs] So it’s pretty much at home, you know we have the laptop.
Mexican American	Yes, a desktop…but my son has an iPad and that is much easier to search. So when I don’t have time to sit down at the computer because someone is using it, well I use that.

**Table 7:** Diabetes knowledge post exposure to website controlling for health literacy, ethnicity, transtheoretical model stage for exercise and fruit and vegetable consumption, and Internet literacy.

Parameter	Estimate	Standard Error	*P* value
Intercept	10.78	4.77	.027
Pre-diabetes knowledge score	0.40	0.07	<.001
Health literacy	0.13	0.14	.37
African American	-0.84	0.85	.32
Caucasian	0.81	0.77	.30
Mexican American	0.00	.	.
Pre-exercise stage			.48^b^
Pre-exercise stage CONTEMPLATION	-0.29	0.85	.74
Pre-exercise stage PREPARATION (REFERENT)	0.00	.	.
Pre-exercise stage ACTION	0.75	0.74	.32
Pre fruit/vegetable stage			.010^b^
Pre fruit/ vegetable stage PRE-CONTEMPLATION	-3.09	0.90	001^a^
Pre fruit/ vegetable stage CONTEMPLATION	-0.21	0.75	.78
Pre fruit/ vegetable stage PREPARATION	0.00	.	.
Pre fruit/ vegetable stage ACTION	-1.073	1.87	.57
Internet literacy score	-0.04	0.02	.04^a^

^a^Significant at *P*≤.05.

^b^From *F* test

**Table 8:** Intention to eat a healthy diet post exposure to website controlling for health literacy, ethnicity, transtheoretical model stage for exercise and fruit and vegetable consumption, and Internet literacy.

Parameter	Estimate	Standard Error	*P* value
Intercept	3.03	1.20	.01
Pre-intention for healthy diet	0.51	0.09	<.001
Health literacy	-0.01	0.03	.78
African American	-0.26	0.19	.19
Caucasian	-0.20	0.18	.29
Mexican American	0.00	.	.
Pre-exercise stage			.073^b^
Pre-exercise stage CONTEMPLATION	-0.17	0.20	.41
Pre-exercise stage PREPARATION (REFERENT)	0.00	.	.
Pre-exercise stage ACTION	-0.41	0.18	.023^a^
Pre fruit/ vegetable stage			.62^b^
Pre fruit/ vegetable stage PRE-CONTEMPLATION	0.05	0.22	.82
Pre fruit/ vegetable stage CONTEMPLATION	-0.12	0.18	.50
Pre fruit/ vegetable stage PREPARATION	0.00	.	.
Pre fruit/ vegetable stage ACTION	0.44	0.44	.32
Internet literacy score	-0.01	0.01	.30

^a^Significant at *P*≤.05

^b^From *F* test

## Discussion

### Principal Findings

This study examined health information seeking on the Internet for African Americans, Caucasians, and Mexican Americans as well as learning and intention changes related to exposure to a diabetes prevention/-education website. Qualitative data describe seeking, access, and use of Web-based health information. Quantitative pre-post measures document an association between exposure to health content on a website and changes in diabetes knowledge and intention to eat a healthy diet.

Using a mixed method approach, we provide insight by ethnic group that may be helpful to other developers of Web-based health content. We found that health information is sought commonly across ethnic groups, but that diabetes-related information is less commonly sought even among those at risk. We also found that despite risks, individuals react emotionally to information showing their risk categorization. These emotions ranged from surprise and concern to not surprised and encouraged. Our participants recalled information from the website on physical activity and healthy food choices that could help them prevent diabetes, but not all indicate intention to act on this information. We also showed that in past Internet searches, only African Americans reported seeking nutrition-related information—one part of the energy expenditure equation that if improved could prevent the development of diabetes. This diabetes prevention site shows promise for promoting behavior change. Other eHealth interventions have also shown support for behavior change ranging from the promotion of physical activity and/or proper nutrition among a workforce [30,31], to information about influenza and the common cold [28], to breastfeeding education [32], to education about mammography aimed at Taiwanese women [33], to patients suffering from depression [34] and schizophrenia [27].

This study documents that while all three populations do access the Internet, Caucasians do so more often with mobile devices at this point in time. The Caucasian participants in this study also reported greater education and income than did the African American and Mexican American participants. This is a limitation of this study and seems to corroborates other studies’ results indicating that socioeconomic status and Internet access are more strongly linked to online health information seeking behavior than is race or ethnicity alone [11,35,36]. However, we also did have a diverse array of electronic devices reported for accessing the Internet across ethnic groups (tablets, phone, laptops, and desktops). In the future, Web-based health information should be prepared on platforms for mobile devices as people are moving more to cell phones and tablets to access the Internet instead of being bound to desktop computers.

Several features of the site provided useful information to the participants across ethnicity. The diabetes risk calculator based on the qualitative responses from participants provided an emotive reaction, useful in fostering behavior change [37,38]. Additionally, based on quantitative results, exposure to the website was associated with changes in intention to make healthier food choices. Also, exposure to the website was associated with increased knowledge about diabetes.

At least two points need to be made about improvements to this website, which may also be informative for other health promotion website sites. The design of diabetes prevention and control websites to accommodate low eHealth literacy with particular attention on learning and usability issues [14] across ethnic populations is an area of growing importance. While there were several features of the site that were designed with low eHealth literacy populations in mind (eg, explanatory videos with lay role models), those with lower eHealth literacy showed less intention to apply the healthful food choice information available on the site. Future research should consider how best to obtain formative feedback through engagement of low eHealth literacy populations and then more effectively pilot test the content and activities designed to meet their needs.

The second point is related to the design of websites for increasing physical activity. Our findings further demonstrate that Web-based information has the potential to reach diverse populations with information and activities that can increase physical activity. However, this study found that exposure to the website was not associated with increased intention for physical activity. Like kinetic video gaming that has advanced to promote physical activity, website developers should explore innovative strategies for going beyond knowledge enhancement to addressing intention and actual energy expenditure among those who visit their site.

We found the overall expected return of 81% was consistent with other studies that did track use [39], or return rates, over an extended period of time, where return rates of between 88% [40] and 77% [31] were reported, and the likelihood of participants to return to the site generally decreasing with time. Returning to a site, particularly when it can support changes in energy expenditure over time is an important feature to explore among those at risk for diabetes.

### Limitations

There are limitations to the study including its small sample size and the lack of a control group not exposed to the website. From the qualitative data collected in the three populations we reached theoretical saturation where no new conceptual ideas were being presented during the interviews. We were able to determine saturation through our simultaneous data collection and analysis process. With a sample size of 71 participants, we discontinued enrollment. From a quantitative perspective, this is a small sample size, and yet we were able to detect several statistically significant findings. Future research should expand the quantitative portion of this research to more fully explore the knowledge, intention for behavior change, and Transtheoretical Model Stage of Change outcomes.

Another limitation of the study was the lack of a control group that completed identical pre- and posttest measures but was not exposed to the website. Budgetary limitations and a prioritization of enrollment of three ethnic groups determined the study design. One way we tried to control for potential biases associated with this design was the immediacy of the posttest measures (directly following website viewing). In this way there was more, although not complete, control of what intervention content could account for any changes in knowledge and behavioral intention.

Future evaluation of the websites should directly address these two limitations. The sample size for Caucasian, African American, and Mexican American adults should be increased to allow for robust statistical analysis with the power to detect statistically significant differences. Additionally, the evaluation should include a control group that is exposed to a comparable website with diabetes information (eg, American Diabetes Association or Centers for Disease Control and Prevention).

### Conclusions

The results of this study advance the field of eHealth diabetes prevention on two fronts. The qualitative results provide insight into the seeking, access, and use of Web-based health information across three ethnic groups in two languages. Additionally, this study provides evidence of an association found between exposure to Web-based health content and related changes in diabetes knowledge and intention to eat a healthy diet.

## Multimedia Appendix 1

Diabetes home page in English.

## Multimedia Appendix 2

Interactive videos available in Spanish and English.

## Multimedia Appendix 3

Inyectando insulina.

## Multimedia Appendix 4

Theme: Reacting emotionally to risk score.

## Abbreviations

ADA: American Diabetes Association
BMI: body mass index
PRIDE: Pittsburgh Regional Initiative for Diabetes Education
