# Supplementary figures and images for: A Type 2 Diabetes Prevention Website for African Americans, Caucasians, and Mexican Americans: Formative Evaluation
Source: JMIR Res Protoc. 2013 Jul 11;2(2):e24. doi: 10.2196/resprot.2573 (PMC3713918; doi:10.2196/resprot.2573)

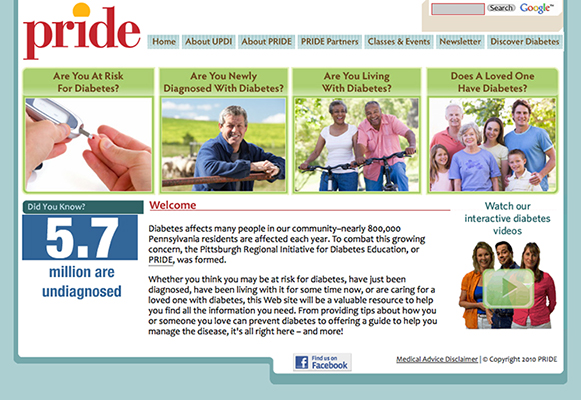

Supplement: Supplementary file 1 [file resprot_v2i2e24_app1.jpg]

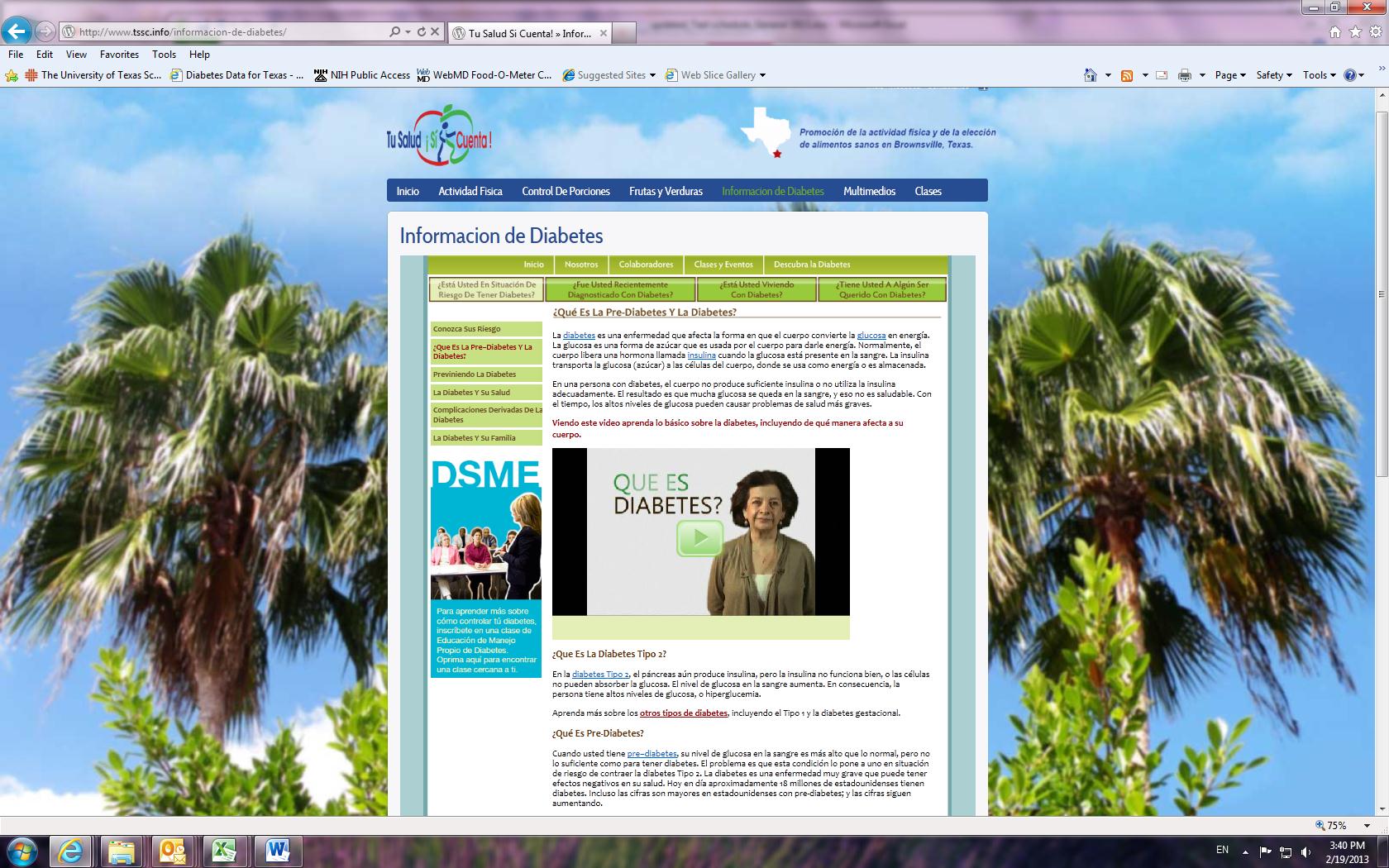

Supplement: Supplementary file 2 [file resprot_v2i2e24_app2.jpg]
