# Supplementary material for: A Type 2 Diabetes Prevention Website for African Americans, Caucasians, and Mexican Americans: Formative Evaluation
Source: JMIR Res Protoc. 2013 Jul 11;2(2):e24. doi: 10.2196/resprot.2573 (PMC3713918; doi:10.2196/resprot.2573)
Supplement: Supplementary file 4 [file resprot_v2i2e24_app4.pdf]

Multimedia Appendix 4. Theme: Reacting emotionally to risk score.

Participant Ethnicity	Surprise
African American	My risk score was moderate, and I am kind of, I guess I'm kind of surprised about that, because I thought it would be high. I mean, both my parents had diabetes, and, you know, I've seen what it can do. It's a really good reminder for me to do something now instead of waiting until tomorrow to do something.
Caucasian	Yeah, it said I was high. That's 'cause I don't exercise and I'm overweight. I was surprised. But I guess that's right 'cause I used to exercise and I don't do it anymore. So I've consequently gained a lot of weight, and I guess I am high. And I'm 52, so, I'm sure I'm high.
Mexican American	My risk score was high and I feel confused, because I know that I'm overweight but I've always checked myself and I've always come out low. I don't have any family that have been diagnosed. My in-laws, yes, but from my family no. I'm more concerned for my children than me, and I come out with a high level.
	Not Surprised
African American	My score was high, but I knew that, so it didn't make me feel any certain type of way…Everybody's diabetic in my family. I'm one of the lucky ones. I have a brother right now in stage-5 kidney failure from complications of diabetes. My dad died from it. Yeah, so it's really high.
Caucasian	I was moderate. I feel I sort of knew it because I need to lose weight, but it helped to have the ways to prevent it and know there are small changes you can make to sort of limit that.
Mexican American	Only said moderate. It didn't say a percentage, or anything. No, no I hadn't heard anything like this but in my family my mom has it, my grandfather has diabetes, and my grandmother also had but she died. And I suppose we run risk of contracting something like that or of developing it.
	Other Emotional Reactions
African American	My risk scores were low, and I feel that's encouraging.
Caucasian	I was moderate. I would say surprised and suspicious. So it made me wonder. Would I go and call my doctor? Maybe.
Mexican American	It scares me…Yes...because I'm beginning to be more conscious in healthy foods and in physical activity. I had done this when I was younger but you stop because it's not about you anymore. It's about your family and your children. And in the end it is contradictory because you must be well so that everyone else is. That is what urges me.
	Risk Score As Motivator For Behavior Change
African American	Well, it looks like it was high, so I'm concerned about that…I am concerned because I'm overweight—you know, I'm a big guy anyway, but I can stand to lose some pounds…it's something that I thought about. Some of the factors,

	like the tiredness. I'm tired, then I'm not exercising. 'Cause I have a job that I walk a lot. So it's like that compensates for exercise, but…not really putting the physical thing into it to really exercise, maybe 30 minutes a day
Caucasian	Well, it came back as high. That didn't thrill me. [chuckles] I mean, 'cause I know I'm overweight and I know it's a risk factor, but I didn't expect it to be high. I thought maybe it was gonna be moderate. So that was eye-opening…I know if I lose a little weight, if I start maybe 30 minutes of walking a day—really helps that a lot. Just like I know my cholesterol's high, and same thing. My PCP said "even if you lose 10% of your body weight, that'll go down."…I just need to get in an exercise routine. And I was exercising in the Fall, but I've gotten complacent now.
Mexican American	No, every time I go to the doctor I check myself. The first thing I ask is to check the sugar because my mom has sugar, my brothers have it…As of right now my sugar has been okay, but I'm trying, right. That topic interests me because my younger daughter of twelve has come out a little high and we had to do several analyses to be sure that she didn't have it. But that doesn't mean she couldn't get it later. So the doctor told me, you have to eat healthy…because I want to live a long life, to help my daughter.
Participant Ethnicity	Risk Score Not Serving As Motivator For Behavior Change
African American	Um, I'm not too pressed about it 'cause I guess according to the body mass index, I'm overweight. [laughs] So I mean, I guess a lot of people are. So, I work out, so I think I should be fine. I'm not like extremely visibly overweight…My doctor hasn't said anything, so… I'm not too concerned.
Caucasian	I was kind of expecting it. I know I don't really take the best care of myself, eating and exercising, and it runs in my family. Both of my parents had diabetes, and I get my eating habits from them. We come from a family that cooks large, unhealthy meals. [chuckles] So I was kind of expecting it…I've never been tested or—I don't have insurance. I haven't seen a doctor in probably four, five years. It's something I expected, but nothing I've really had confirmed.
Mexican American	My dream is it was in the medium [range], but I knew it was going to be high.
